# Acquisition of cancer stem cell capacities after spontaneous cell fusion

**DOI:** 10.1186/s12885-021-07979-2

**Published:** 2021-03-07

**Authors:** Candice Merle, Pauline Lagarde, Lydia Lartigue, Frédéric Chibon

**Affiliations:** 1 INSERM U1037, Cancer Research Center in Toulouse (CRCT), 31037 Toulouse, France; 2 University of Toulouse 3, Paul Sabatier, 118 route de Narbonne, 31062 Toulouse Cedex 9, France; 3 INSERM U1218, 229 cours de l’Argonne, F-33076 Bordeaux, France; 4 University of Bordeaux, 146 rue Léo Saignat, F-33000 Bordeaux, France; 5 Institut Claudius Régaud, IUCT-Oncopole, Toulouse, France; 6 Present address: CRCT-IUCT-O, 2 avenue Hubert Curien, 31037 Toulouse Cedex 1, France

**Keywords:** Cell fusion, Cancer stem cells, Sarcoma

## Abstract

**Background:**

Cancer stem/Initiating cell (CS/IC) hypothesis argues that CS/ICs are responsible of tumour initiation, drug resistance, metastasis or disease relapse. Their detection in several cancers supports this concept. However, their origin is still misunderstood. Cell fusion is shown to take part in the formation of CS/ICs, i.e. fusion between mesenchymal stem cell and cancer cell. In a previous paper, we described that fusion leads to hybrids with metastatic capacity. This process triggered genomic rearrangements in hybrid cells together with increased metastasis development. Here, we hypothesize that cell fusion could be strong enough to provoke a cellular reprogramming and the acquisition of CS/IC properties, promoting metastasis formation.

**Methods:**

After spontaneous cell fusion between E6E7 (IMR90 with the oncogenes E6 and E7) and RST (IMR90 fully transformed) cell lines, hybrid cells were selected by dual antibiotic selection. Cancer stem cells capacities were evaluated regarding capacity to form spheres, expression of stem cell markers and the presence of ALDH*high* cells.

**Results:**

Our data show that after cell fusion, all hybrids contain a percentage of cells with CS/ICs properties, regarding. Importantly, we lastly showed that NANOG inhibition in H1 hybrid decreases this migration capacity while having no effect on the corresponding parental cells.

**Conclusions:**

Altogether these results indicate that the combination of CS/ICs properties and genomic rearrangement in hybrids is likely to be key to tumour progression.

**Supplementary Information:**

The online version contains supplementary material available at 10.1186/s12885-021-07979-2.

## Background

A century ago, Aichel formulated the hypothesis that cell fusion (macrophage/cancer cell) could be at the origin of metastatic cells. From that date, several papers have demonstrated that diverted cell fusion could be a process leading to the development of tumours and metastasis. Cell fusion occurs at different stages of tumour development from initiation to the development of metastasis [1, 2]. Cell fusion events were detected in animal model [3, 4] and in human tumours [5–7]. Furthermore, Gast et al. recently identified hybrids in the blood of pancreatic cancer patients, and their presence is associated with a poor prognosis [3]. These data highlight the importance of cell fusion and hybrid cells in the mechanism of dissemination. However, the regulation of cell fusion in tumour and the consequences for the hybrid are still misunderstood.

Tumours present heterogeneous cancer cell population with genetic and phenotypic differences. To explain this diversity, many models were established. The model of clonal evolution presents that the heterogeneity arises through stochastic genetic and epigenetic changed that confers heritable phenotypic differences upon cancer cells. Then, the fittest cells will be selected in the tumour following a Darwinian evolution [8]. A second model, described a hierarchical organisation of tumours, where cancer stem/initiating cells (CS/ICs) are at the top of the pyramid [9, 10]. CS/ICs have an unlimited capacity for self-renewal and can also generate non CS/ICs progeny (differentiated progeny) [11]. Their origin is still an ongoing debate but two main hypotheses hold sway. First, CS/ICs could arise from normal stem cells that accumulate mutations leading to transformation. Second, differentiated cells acquire mutations that lead to reprogramming [12]. Fusion events may also be involved in the generation of CS/ICs. In fact, fusion between normal stem cells such as mesenchymal stem cells and bone marrow-derived stem cells, on the one hand, and cancer cells on the other, leads to the formation of hybrids with tumorigenic and stemness properties, i.e. the hallmarks of CS/ICs [13–15]. Furthermore, CS/ICs exhibit stem cell markers (OCT4 and NANOG) and have an increased metastatic capacity [16].

Metastasis is a complex and still only partially understood process. Several studies have described the acquisition of metastatic capacity at the early stage of tumour development, after a burst event leading to invasive cancer and metastasis [17–19]. In addition, several papers demonstrate that CS/ICs are composed of different clones, of which some known as metastasis-initiating cells (MIC) could have the capacity to metastasise [20, 21]. However, how CS/IC become MIC is still not understood.

In a previous study, we demonstrated that spontaneous fusion between IMR90 E6-E7 (E6E7) fibroblasts with IMR90 E6-E7- HRAS_G12V_ -SmallT-hTERT (RST) fibroblasts is a burst event leading to cellular heterogeneity and the acquisition of metastatic capacity by hybrids [22]. The goal of this paper was to test, in that model, the hypothesis that cell fusion triggers the acquisition of CS/IC properties, leading to the development of metastatic capacity.

## Results

### Fused cells exhibit ALDH*high* activity immediately after cell fusion

As previously described [22], hybrid cell lines (H1 to H4) were established after spontaneous cell fusion of E6E7 (IMR90 immortalized with E6 and E7) and RST (IMR90 transformed with E6, E7, RAS, Smallt and hTERT) (fusion event represents less than 1% of the population). RST and hybrid cell lines developed Undifferentiated Pleomorphic Sarcomas after subcutaneous injection in mice. However, hybrid cell lines possess an increased migration capacity in vitro and a higher metastatic capacity (absent in parental cell lines) [22].

**Fig. 1:**
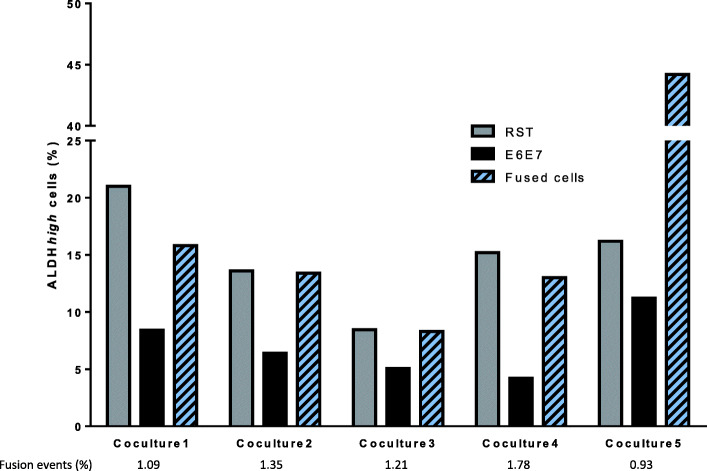
Percentage of ALDH*high* cells in parental cell lines and fused cells after 72 h of coculture, evaluated by flow cytometry

Elevated aldehyde dehydrogenase (ALDH) activity is considered a suitable marker for the identification of normal and cancer stem cells, as defined by ALDH*high* cells [23, 24]. Cell populations with high ALDH activity has been detected in many sarcoma histotypes and enabled the identification of CS/ICs [25–27]. To validate this marker in our model, parental and hybrid cell lines were cells sorted according to their ALDH activity (ALDH*low* versus ALDH*high* cells) (Fig. S1 A). They were sorted and plated in conditions to grow as spheres. The frequency of CS/ICs in these two populations was determined by Extreme Limiting Dilution Analysis (ELDA) method [28] (Fig. S1 B). The frequency of CS/IC ranges from 0 to 1/873 in ALDH*low* cells and from 1/3203 to 1/82 in ALDH*high* cells. For all the cell lines except E6E7, the frequency of CS/ICs is significantly enriched in ALDH*high* populations. This confirms that ALDH activity is a consistent marker of CS/ICs in this cell line model. Hence, we used this measured ALDH activity to quantify CS/ICs in parental cell lines and hybrids. ALDH*high* cell percentage was evaluated after 72 h, the time to get spontaneous hybrids, of coculture of RST and E6E7 (Fig. 1, Fig. S2) for RST cells (DsRed+ cells), E6E7 cells (CFP+ cells) and hybrids cells (DsRed+/CFP+ cells).

The experiment was repeated three times and each time in one co-culture, hybrids cells had a percentage of ALDH*high* cells two-fold higher than in the other samples (co-culture 5 in the experiment represented). Since parental and fused cells both presented the CS/IC marker, we wondered whether it was due to the transmission or acquisition of CS/IC properties following cell fusion.

### Identification of a population with stem cell properties in hybrid cell lines

**Fig. 2:**
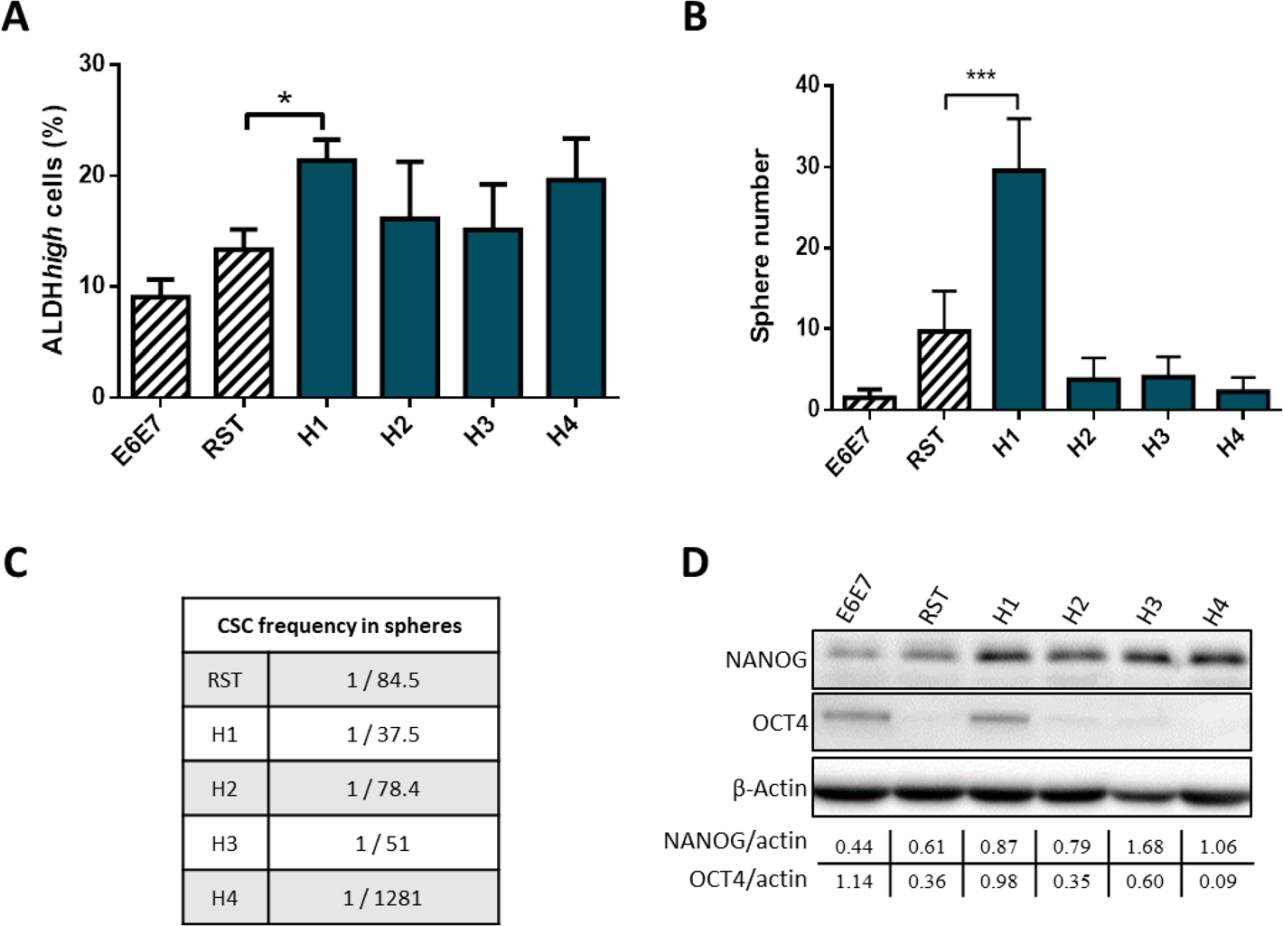
**a.** Percentage of ALDH*high* cells in parental and hybrid cell lines evaluated by flow cytometry. * *p* value < 0,05; *n* = 4. **b.** Number of spheres after 10 days of culture in non-adherent condition. *n* = 6 **c.** Evaluation of stem cell frequency by ELDA method in spheres formed to evaluate self-renew capacity. **d.** Expression of NANOG and OCT4 by western blotting analysis in the monolayer culture of each cell lines. β-actin was used as loading control. Full-length blots are presented in Supplementary Fig. 4

To assess the presence of CS/ICs in parental and hybrid cell lines, several CS/IC markers were tested in the hybrid clones and parental cell lines. First, we sought to detect ALDH*high* cells. We found that they were present in all parental and hybrid cell lines (Fig. 2 a), and that this phenotype persisted in all passages. Their percentage was not significantly different between RST, H2, H3 and H4.

However, their population was higher in H1 than in the parental cell lines E6E7 and RST. In non-adherent condition and SVF-free medium at day 10, all cell lines had developed spheres (Fig. 2 b, Fig. S3 B). To further investigate their self-renewal capacities, these spheres were dissociated at day 12 when they were completely formed, and were re-plated in a 96-well plate ultra-low attachment. Except for E6E7 in which the number of spheres was too low to complete the experiment, all cell lines demonstrated the formation of secondary spheres at a frequency ranging from 1/37.5 (H1) to 1/1281 (H4) (Fig. 2c).

To go further, the CS/IC population was also evaluated by the detection of two stem cell markers: NANOG and OCT4 [28]. NANOG was expressed in parental and hybrid cell lines, with a stronger expression in all hybrid cell lines (Fig. 2d, Fig. S4). OCT4 expression was weak in RST, H2 and H3, higher in E6E7 and H1, and not detected in H4. Hybrids H1, H2 and H3 thus have a strong NANOG expression with a moderate (H1) or weak (H2 and H3) OCT4 expression. The parental cells have a moderate NANOG expression with a moderate (E6E7) or weak (RST) OCT4 expression.

To sum up, RST and hybrid cell lines have a population of cells with the capacity to form spheres and to self-renew, to express stem cells markers, and possessing a high ALDH activity. Therefore, RST and hybrid cell lines contain CS/IC population. Their proportion seems to be identical between RST and H2, H3 and H4. Only H1 has a higher percentage of CS/ICs.

### Generation of ALDH*high* cells after cell fusion

**Fig. 3:**
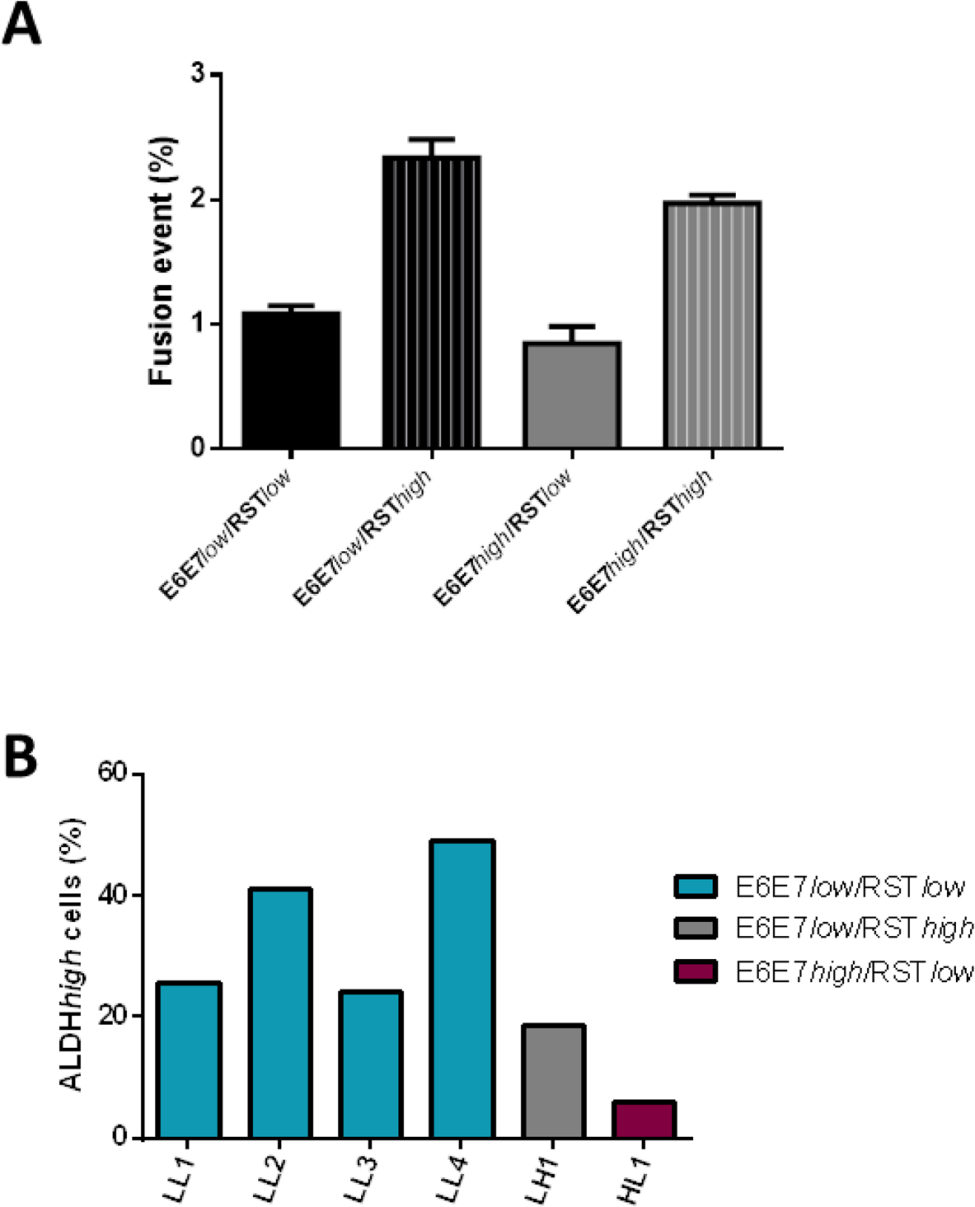
**a.** Percentage of cell fusion with different combinations of cell culture according to ALDH activity (*n* = 3). Percentages were not statistically different. **b.** Percentage of ALDH*high* cells in clones derived from spontaneous cell fusion

Because a population of cells in RST and all hybrid cell lines were found to possess CS/ICs properties, we investigated whether these properties are generated upon cell fusion or is transmitted from RST to the new hybrid. E6E7*low* were thus cocultured for 72 h with RST*low* or RST*high* and E6E7*high* with RST*low* or RST*high*. After 72 h of co-culture, cell fusion events were counted by cytometry (Fig. 3a, Fig. S5).

A higher frequency of fusion events was observed with the combination E6E7*low*/RST*high* and E6E7*high*/RST*high*. After co-culture, dual antibiotic selection was added to select hybrids. Four clones were established in the E6E7*low*/RST*low* condition, one clone each with E6E7*low*/RST*high* and E6E7*high*/RST*low,* and none with E6E7*high*/RST*high*. The percentage of ALDH*high* cells was determined in all these hybrid clones. Interestingly, ALDH*high* cells (Fig. 3b) were detected in all clones, ranging from 5.9% (HL1) to 49% (LL4). Hence, the fusion of negative ALDH cells may generate ALDH*high* cells.

### Inhibition of NANOG decreases migration capacity in hybrid

**Fig. 4:**
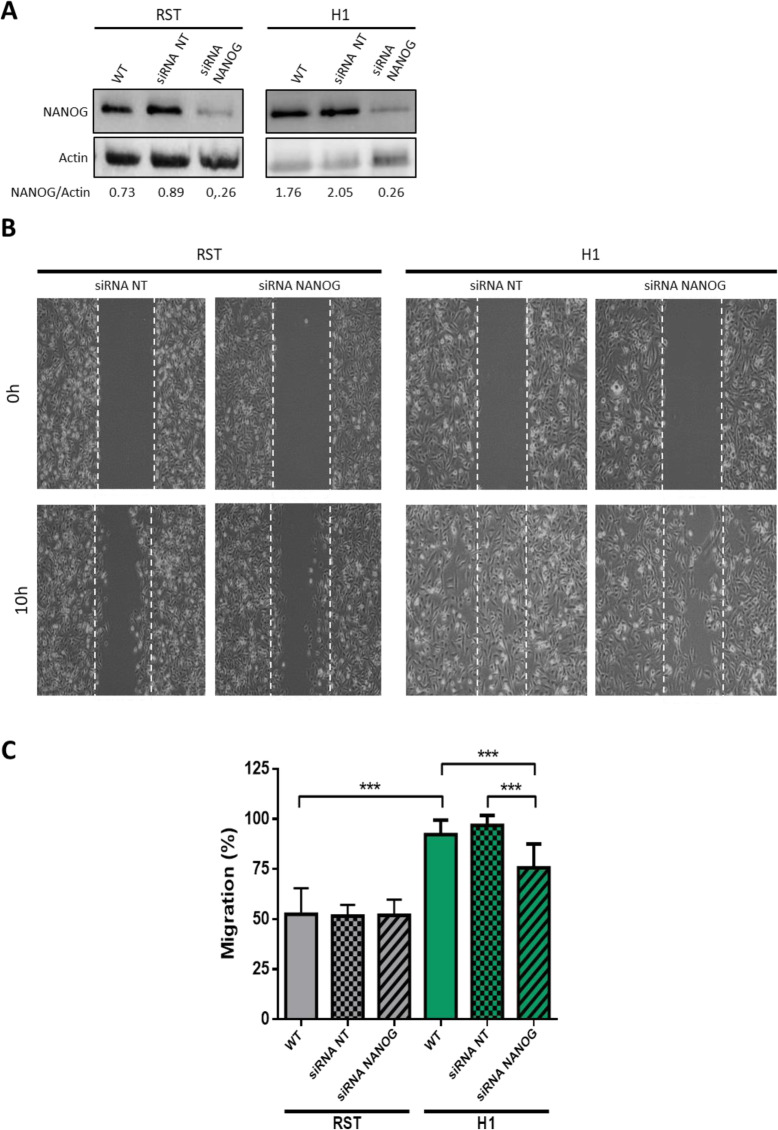
**a.** At the top, **e**xpression of NANOG and β-actin evaluated by western blotting in wild type cell lines and after 72 h of incubation with siRNA negative control or siRNA NANOG. At the bottom, quantification of NANOG expression normalized by β-actin expression (loading control). Full-length blots/gels are presented in Supplementary Fig. 6. **b.** Images of scratch test migration assay at 0 h and 10 h after inhibition of NANOG by siRNA compared to negative control siRNA. **c.** Evaluation of migration with scratch test for RST and H1 in wild type cell lines and after incubation with non-targeted siRNA or siRNA NANOG. *** *p* value < 0.001 (Mann-Whitney test) (*n* = 16)

As previously described [22], H1 migrated significantly more than RST. To determine whether CS/ICs are involved in the increasing migration capacity of the hybrid, NANOG, which was strongly expressed by all hybrids, was inhibited by siRNA (Fig. 4a) and migration capacity was evaluated (Fig. 4b, c).

RST with siRNA against NANOG did not modify migration compared to RST WT and RST with non-targeted siRNA. Contrasting, inhibition of NANOG in H1 significantly decreased its migration capacity compared to the negative control (Fig. 4c). RST and H1 both contain CS/ICs. However, the inhibition of the principal marker of this population does not have the same impact in the parental and in H1 hybrid. Unlike RST CS/ICs, CS/ICs from H1 can migrate in vitro and this capacity is NANOG-dependent.

## Discussion

In the present study, we tested the hypothesis that the tumour aggressiveness and metastatic capacity acquired by hybrids [22] could be associated with the stemness phenotype developed by hybrids upon fusion. The results show that all hybrid cell lines had a population of cells with stemness properties. The percentage of CS/ICs remained stable during the passages. However, these CS/IC populations did not demonstrate the same capacity to grow as spheres, the same expression of *OCT4* and *NANOG* or the same percentage of ALDH*high* cells. For example, H1 highly expressed *NANO*G and *OCT4* whereas the other hybrids expressed *NANOG* similarly but weakly expressed *OCT4*. H1 also had a higher percentage of ALDH*high* cells and a greater capacity to develop spheres. Fusion between E6E7 and RST led to the formation of hybrids with heterogeneous properties, as metabolism and in vitro migration and invasion capacity [22] but were all able to develop metastases in mice. Since cell fusion is a driver of cell diversity and heterogeneity, we hypothesise that the number of hybrid cells with stemness properties is different in each hybrid, and that their own properties are also different.

Hybrid cells with CS/IC properties have been shown to form after fusion between stem cells (normal or tumoral) and differentiated cells [13, 29–32]. In the present study, the fusion of E6E7 ALDH*low* and RST ALDH*low* led to the formation of a population of cells with ALDH*high* activity. ALDH activity is known to be a valuable marker of CS/IC populations in soft tissue sarcomas [26–28]. While in vivo experiments remain to be done because limiting dilution in mice is the golden standard to demonstrate the presence of CSCs, these data together with previous publications show that cell fusion can produce a hybrid with stemness capacity, even if neither parent possesses it. We and others have already shown that cell fusion involves massive genomic reorganization [1, 33–35]. Such alterations disturb the expression of several genes and pathways. Over-expression of genes involved in stem cell maintenance, self-renewal and pluripotency, as well as the down-expression of genes associated with differentiation, could lead to reprogramming towards cells with stemness properties. Cell fusion might also modify epigenetic markers that unlock the expression of stemness markers like NANOG and OCT4, which are directly involved in cell reprogramming [36].

The inhibition of NANOG leads to the decrease of H1 migration. Even though our study lacks an in vivo experiment with stable knockdown, our results correlate with data already reported in bladder, ovarian and breast cancer [37–39]. NANOG is known to be a marker of poor prognosis in several cancers [40–42]. This transcription factor is not only a cancer stem cell marker; it also promotes important characteristics of CS/IC such as drug resistance, cancer cell motility and tumour metastasis [43]. NANOG positively regulates MMP-2 and MMP-9, which are factors involved in migration and metastasis [37, 44]. Interestingly, in the present fusion model, the inhibition of NANOG had an impact only on the hybrid cell line and not on the parental RST. Because the inhibition of NANOG impacts cells with stemness properties [45], we hypothesise that CS/IC populations are different in parental and hybrid cell lines. In fact, only cells surviving fusion and having extra chromosomes, genomic rearrangements, epigenetic modifications and stemness properties can become “super” cells able to disseminate and metastasize.

## Methods

### Cell lines and hybrid generation

Cell lines, hybrid cell selection and culture conditions were already described [22]. Parental cell lines were as follows: 1) E6E7 was IMR90 (human foetal lung fibroblast) partially transformed, harbouring two human papilloma viral ONC E6 and E7, targeting p53 and pRB respectively; 2) RST was IMR90 fully transformed harbouring the ONC HRAS G12V, SV40 small T, and hTERT in addition to E6 and E7. These cell lines were established according to the model described by Hahn et al. [46, 47] and were kindly provided by Martin Teichmann. After 72 h of coculture of E6E7 and RST, hybrids were selected by double antibiotic selection (puromycin and blasticidin; Life Technologies, Thermo Fisher Scientific). Fluorescence and genomic analysis were performed to validate the selection of cells from fusion.

### Sphere formation assay

Cells were cultured in suspension in Ultralow Attachment (ULA) plate in DMEM/F12 supplemented with N2-supplement (ThermoFisher). Every three days 10 ng/ml of EGF (R&D systems) and bFGF (ThermoFisher) were added. In a 6-well plates 10,000 cells were plated and after 10 days the number of spheres was manually counted.

To assess stem cell frequency in formed spheres, spheres were dissociated using trypsin and mechanical dissociation. Then, cells were counted and 500–250–100-10 cells / 100 μl were seeded in a 96-well plate with ultra-low attachment. The presence of spheres were determined manually after 10 days and results were analysed with the ELDA method [48].

### Protein extraction and Western blotting

This section was performed as already described [34]. Cells were rinsed in PBS 1X and lysed for 20 min at 4 °C in RIPA buffer (R0278, Sigma Aldrich) supplemented by phosphatase/protease inhibitor cocktail (1X, Sigma Aldrich). Proteins were collected in supernatant after 15 min of centrifugation at 13000 g and quantified by DC protein assay kit, Biorad. On Mini Proteane TGX stain free 4–15% (Biorad), 40 μg of total proteins were loaded. Transfers to PVDF membrane were performed using a dry transfer system (iBlot2, ThermoFisher Scientific) and membranes were blocked in 5% non-fat dry milk in 0.1% PBS-Tween solution. Membranes were incubated overnight with the primary antibody: mouse anti-NANOG (GTX627421, Genetex, 1/200), rabbit anti-OCT4 (653,702, Biolegend, 1/200) and mouse anti-β-actin (A5316, Sigma Aldrich, 1/1000) at 4 °C overnight. After washing, membranes were incubated for 1 h at room temperature with appropriate secondary antibodies: anti-rabbit HRP (7074S, Ozyme, 1/1000) and anti-mouse HRP (7076S, Ozyme, 1/1000). After incubation with chemiluminescent substrate (ECL Immobilon Western, WBKLS0100, Merck), signals were detected using PXi (Syngene).

### Aldehyde dehydrogenase activity

The ALDFELUOR kit (Stem Cell Technologies, Vancouver, Canada) was used to detect ALDH activity according to the manufacturer’s instructions. Briefly, 500,000 cells in 1 ml Aldefluor assay buffer were stained with 5 μl of Aldefluor reagent and 500 μl were used as a negative control with 10 μl of DEAB. Cells were incubated for 45 min at 37 °C. Flow cytometry was performed with BD LSRFortessa (BD Biosciences, Franklin Lakes, NJ, USA) and analysed with FlowJo software.

For cell sorting, dead cells were excluded based on light scatter characteristics and only DsRed-positive cells and CFP-positive cells were selected for RST and E6E7 respectively. Gates were selected in order to choose the brightest (ALDH*high*) and the dimmest (ALDH*high*) cells compared to DEAB-negative control. Cell sorting was done with FACS Melody (BD Biosciences, Franklin Lakes, NJ, USA).

For the quantification of ALDH*high* cells in hybrids right after cell fusion (Fig. 1), parental cell lines E6E7 and RST were cocultured for 72 h in a 6-well plates. Then, cells were harvested and ALDEFLUOR assay was performed as described above. This experiment was repeated three times, with 5 cocolture for each experiment.

#### siRNA NANOG

Cells were harvested, counted and diluted in order to obtain 125,000 cells per well in a 6-well plate. After 24 h, lipofectamine RNAiMAX (13,778,030, ThermoFisher Scientific) was diluted in OPTI-MEM (31,985,062, ThermoFisher Scientific). A solution of 10 nM of siRNA NANOG (s36649, ThermoFisher Scientific, Waltham, MA, USA) or 10 nM of siRNA negative control (4,390,843, ThermoFisher) was prepared with OPTI-MEM. Lipofectamine and siRNA were slowly mixed and incubated for 20 min at room temperature. Then, these complexes were added to the cells and incubated in culture conditions. After 6 h, medium was changed and replaced by culture medium without antibiotics. Optimal inhibition of NANOG, without changes in negative control compared to wild type cells, was detected at 72 h by western blotting.

#### Scratch test migration assay

This section was performed as already described [22]. For the wound healing assay, 4 × 10^5^ cells were plated onto a 6-well plate. Twenty-four hours later, a strip of cells was removed from the monolayer of cells using a pipette tip. Phase contrast images were acquired with a 10× objective at the time of the scratch and 10 h later using a Nikon Eclipse TS100 microscope. Quantification was done using Image J. Data at 10 h were normalized to the size of the wound at 0 h and results were plotted using GraphPad (La Jolla, CA, USA) software.

##### Statistical analysis

Statistical analysis were performed with GraphPad (La Jolla, CA, USA) software.

## Supplementary Information

**Additional file 1: Figure S1.** A. Cytometry dot plot. ALDH*low* cells in blue and ALDH*high* cells in green. Top: cell line incubated with DEAB as negative control. Bottom: cell line without DEAB. B. Evaluation of stem cell frequency by ELDA method in ALDH*low* and ALDH*high* cells after cell sorting. “-“: absence of CS/ICs. **Figure S2.** Percentage of ALDHhigh cells evaluated in parental and hybrid cell population after 72 h of coculture between E6E7 and RST (*n* = 17). Percentages were not statistically different. **Figure S3.** A. Cytometry dot plot representing cell populations selected for cell sorting. B. Images of spheres developed in non-adherent cell culture conditions; scale bar = 50 μm. **Figure S4.** Full length blots. **Figure S5.** Cytometry dot plot with gates used to determine percentage of fused cells after 72 h of co-culture. x-axis represents CFP fluorescence intensity and y-axis represents DsRed fluorescence intensity. **Figure S6.** Full length blots.

## Data Availability

The datasets used and/or analysed during the current study are available from the corresponding author on reasonable request.
