# Supplementary material for: Acquisition of cancer stem cell capacities after spontaneous cell fusion
Source: BMC Cancer. 2021 Mar 7;21:241. doi: 10.1186/s12885-021-07979-2 (PMC7938600; doi:10.1186/s12885-021-07979-2)
Supplement: Supplementary file 1 — Additional file 1: Figure S1. A. Cytometry dot plot. ALDHlow cells in blue and ALDHhigh cells in green. Top: cell line incubated with DEAB as negative control. Bottom: cell line without DEAB. B. Evaluation of stem cell frequency by ELDA method in ALDHlow and ALDHhigh cells after cell sorting. “-“: absence of CS/ICs. Figure S2. Percentage of ALDHhigh cells evaluated in parental and hybrid cell population after 72 h of coculture between E6E7 and RST (n = 17). Percentages were not statistically different. Figure S3. A. Cytometry dot plot representing cell populations selected for cell sorting. B. Images of spheres developed in non-adherent cell culture conditions; scale bar = 50 μm. Figure S4. Full length blots. Figure S5. Cytometry dot plot with gates used to determine percentage of fused cells after 72 h of co-culture. x-axis represents CFP fluorescence intensity and y-axis represents DsRed fluorescence intensity. Figure S6. Full length blots. [file 12885_2021_7979_MOESM1_ESM.pptx]

## Slide 1
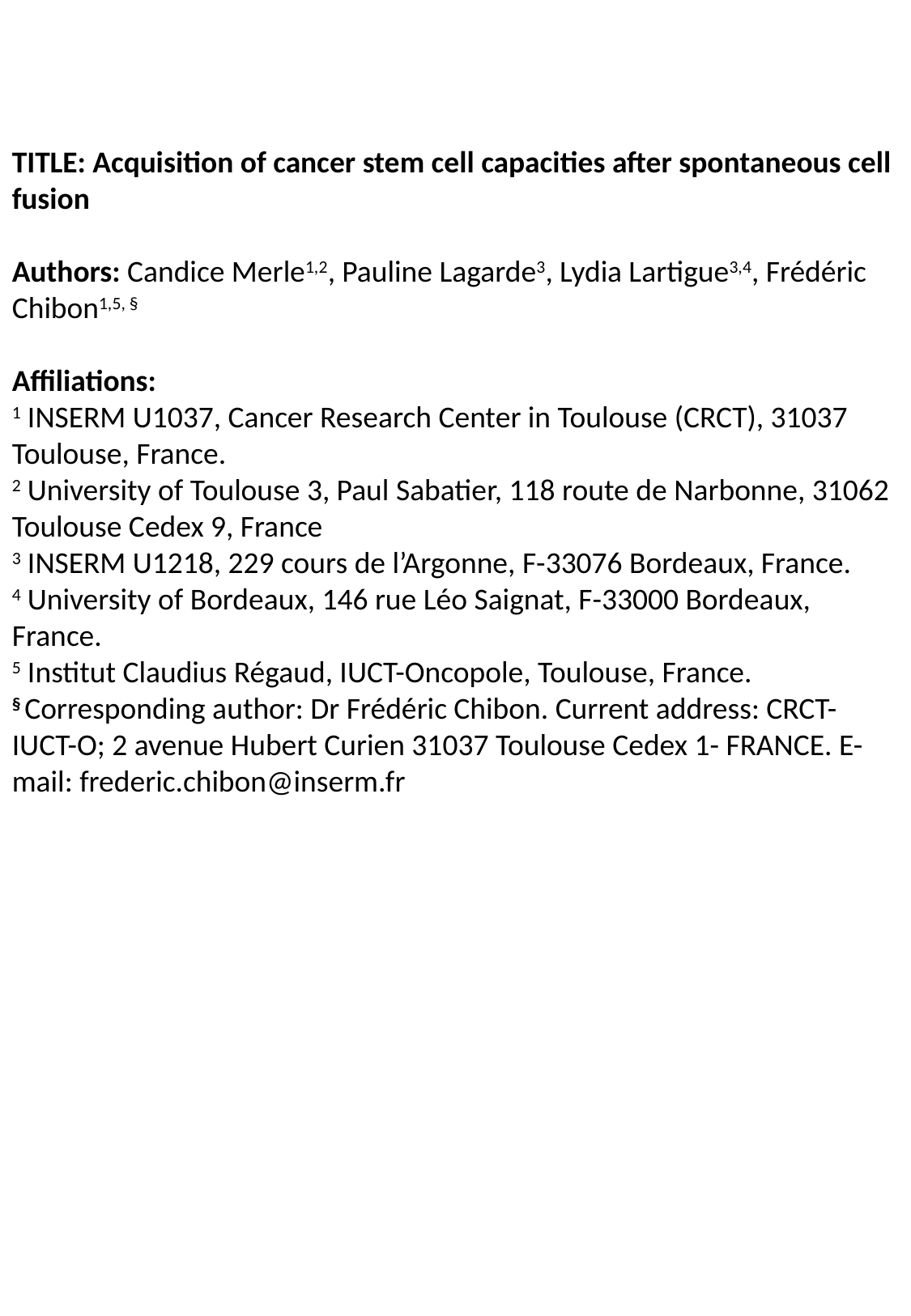

TITLE: Acquisition of cancer stem cell capacities after spontaneous cell fusion
Authors: Candice Merle1,2, Pauline Lagarde3, Lydia Lartigue3,4, Frédéric Chibon1,5, §
Affiliations:
1 INSERM U1037, Cancer Research Center in Toulouse (CRCT), 31037 Toulouse, France.
2 University of Toulouse 3, Paul Sabatier, 118 route de Narbonne, 31062 Toulouse Cedex 9, France
3 INSERM U1218, 229 cours de l’Argonne, F-33076 Bordeaux, France.
4 University of Bordeaux, 146 rue Léo Saignat, F-33000 Bordeaux, France.
5 Institut Claudius Régaud, IUCT-Oncopole, Toulouse, France.
§ Corresponding author: Dr Frédéric Chibon. Current address: CRCT-IUCT-O; 2 avenue Hubert Curien 31037 Toulouse Cedex 1- FRANCE. E-mail: frederic.chibon@inserm.fr

## Slide 2
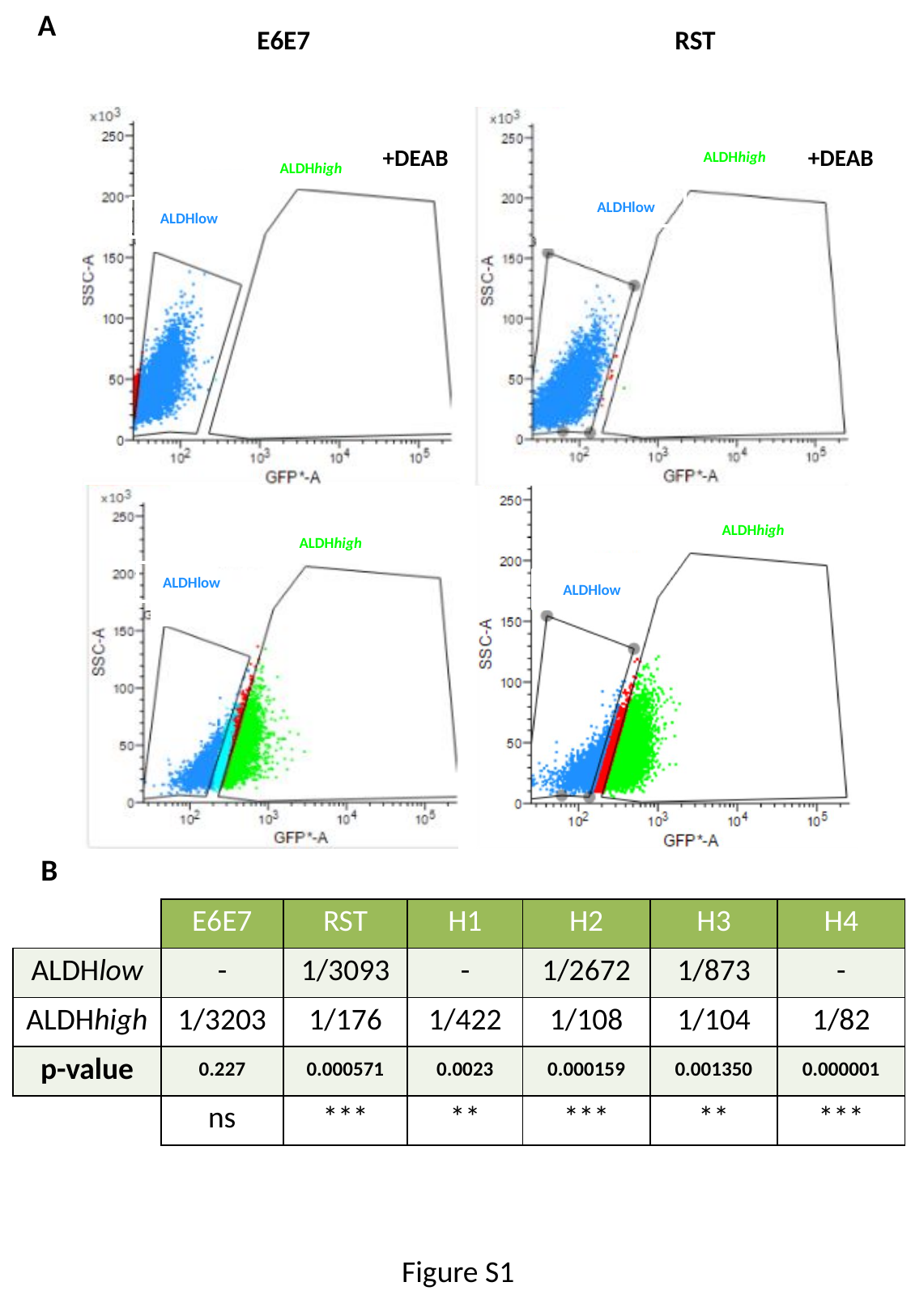

A
E6E7
RST
ALDHhigh
+DEAB
+DEAB
ALDHhigh
ALDHlow
ALDHlow
ALDHhigh
ALDHhigh
ALDHlow
ALDHlow
B
| | E6E7 | RST | H1 | H2 | H3 | H4 |
| --- | --- | --- | --- | --- | --- | --- |
| ALDHlow | - | 1/3093 | - | 1/2672 | 1/873 | - |
| ALDHhigh | 1/3203 | 1/176 | 1/422 | 1/108 | 1/104 | 1/82 |
| p-value | 0.227 | 0.000571 | 0.0023 | 0.000159 | 0.001350 | 0.000001 |
| | ns | \*\*\* | \*\* | \*\*\* | \*\* | \*\*\* |
Figure S1

## Slide 3
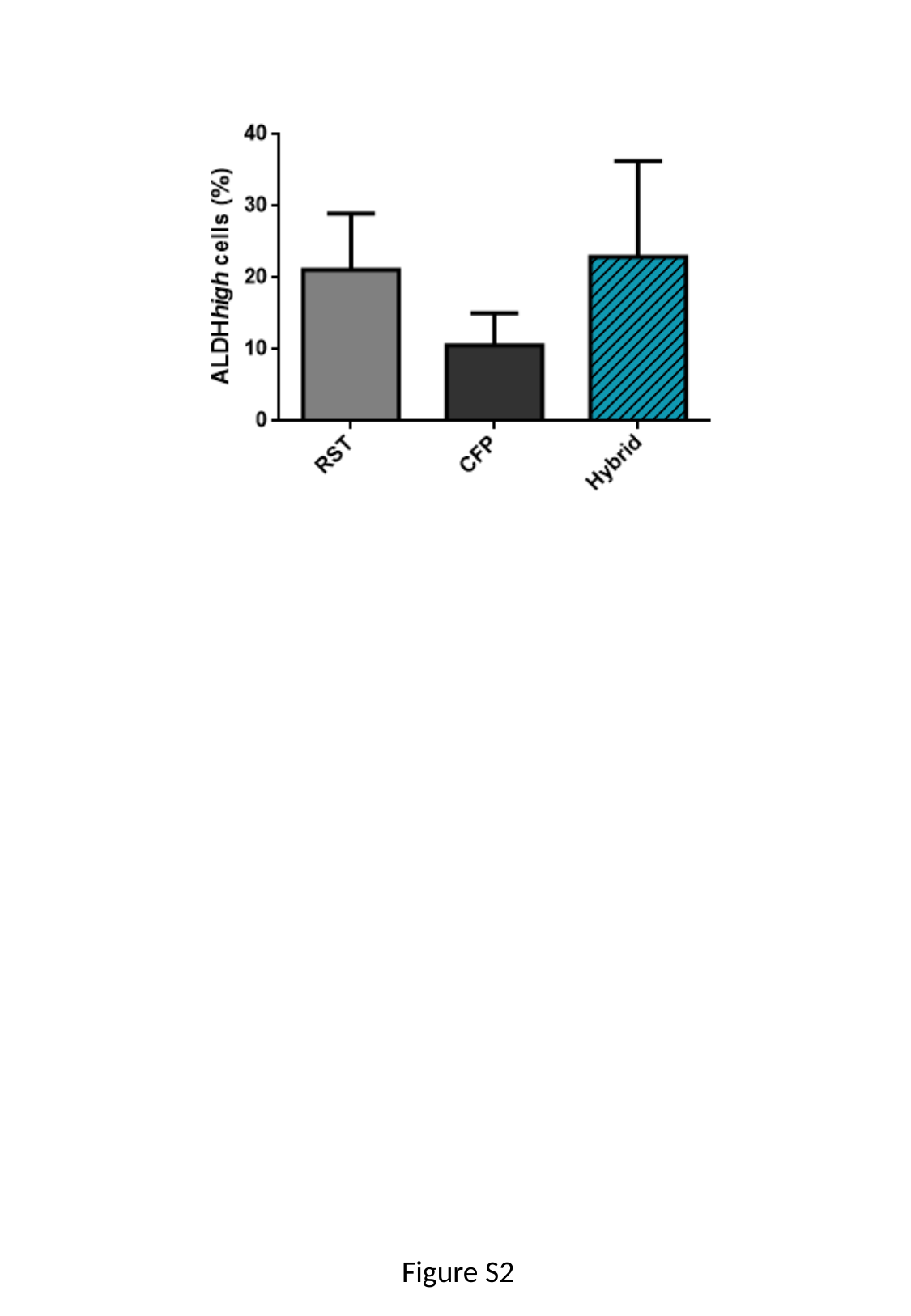

Figure S2

## Slide 4
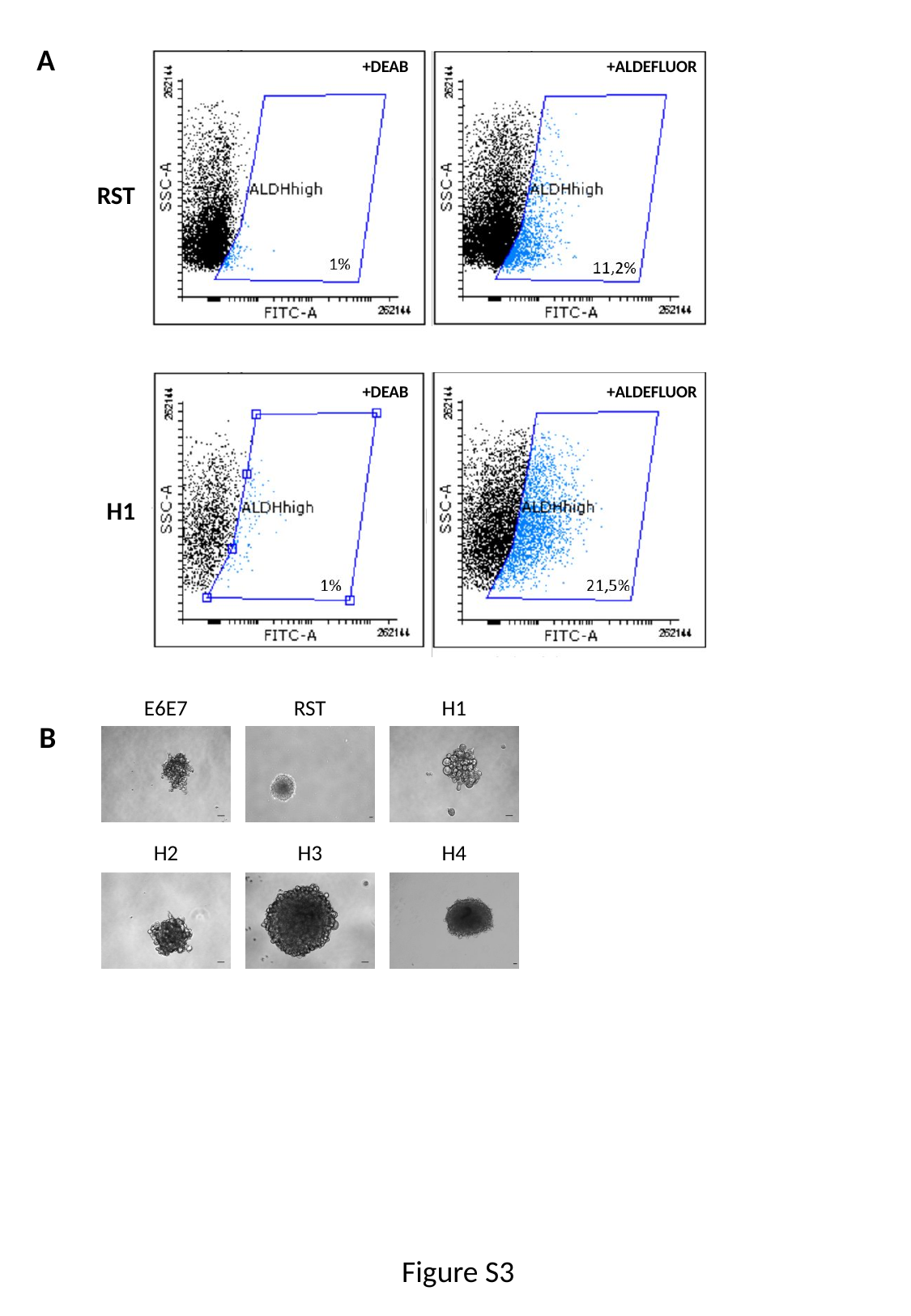

A
+ALDEFLUOR
+DEAB
RST
+ALDEFLUOR
+DEAB
H1
E6E7
H2
RST
H3
H1
H4
B
Figure S3

## Slide 5
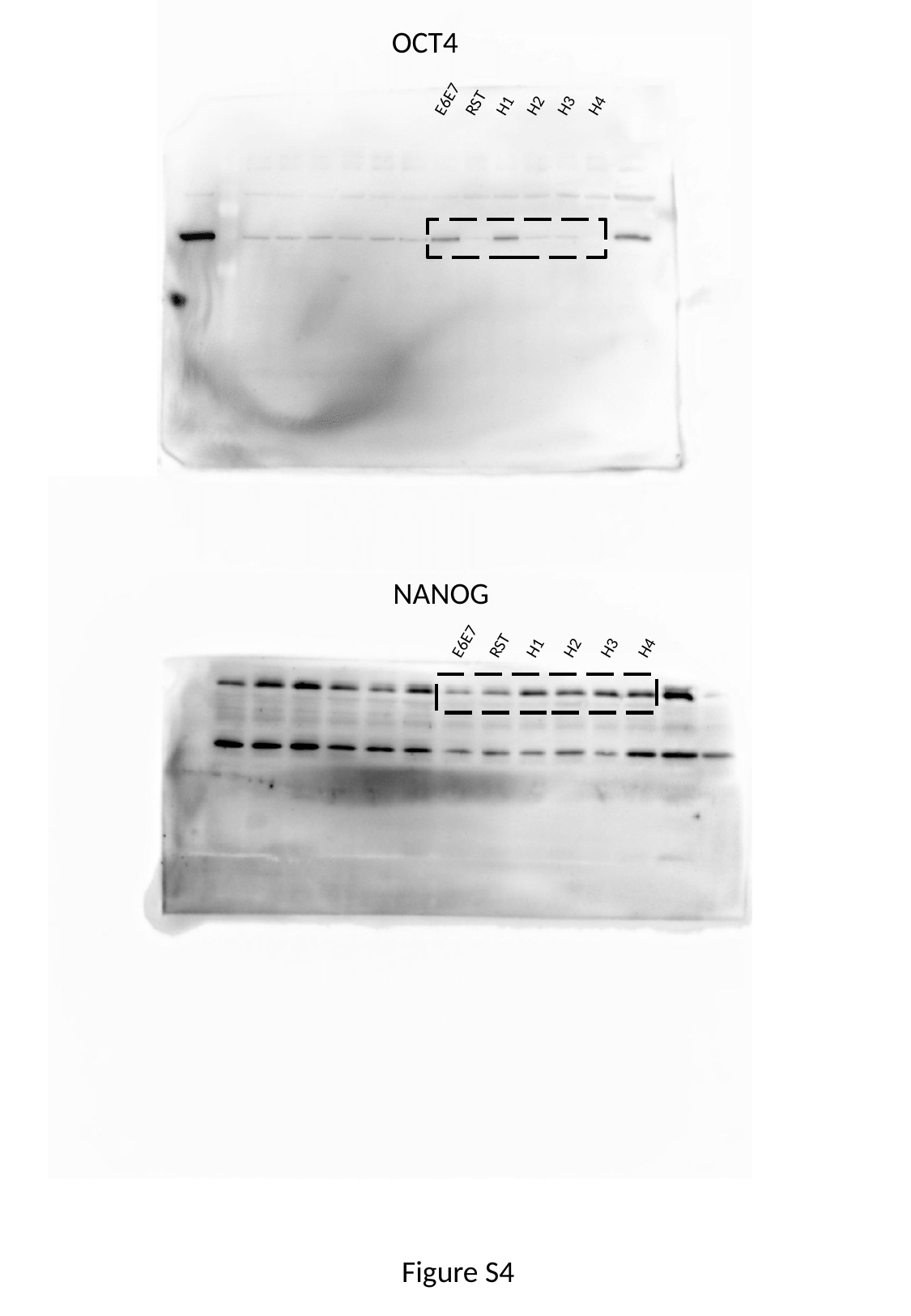

OCT4
E6E7
RST
H1
H2
H3
H4
NANOG
E6E7
RST
H1
H2
H3
H4
Figure S4

## Slide 6
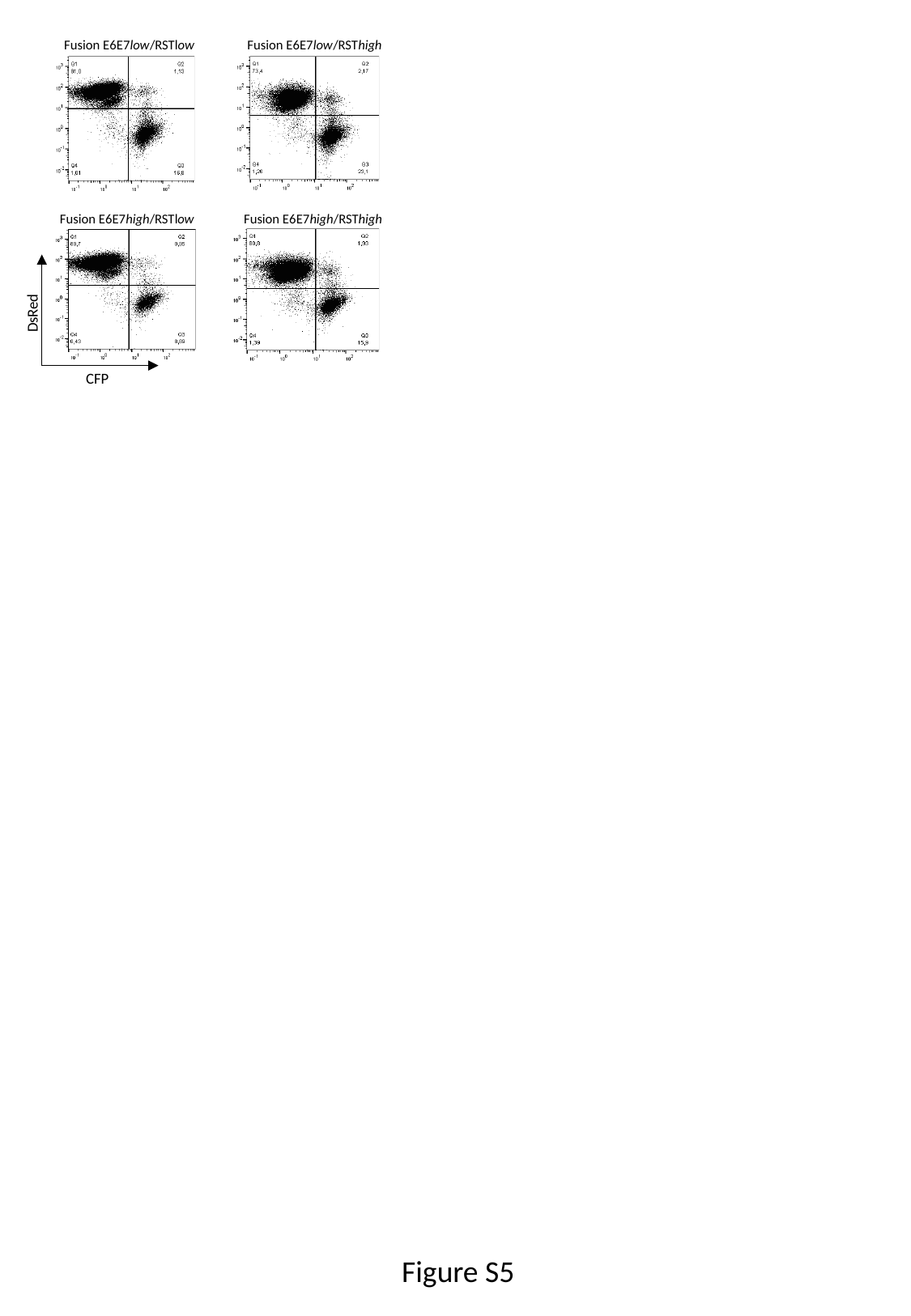

Fusion E6E7low/RSTlow
Fusion E6E7low/RSThigh
Fusion E6E7high/RSTlow
Fusion E6E7high/RSThigh
DsRed
CFP
Figure S5

## Slide 7
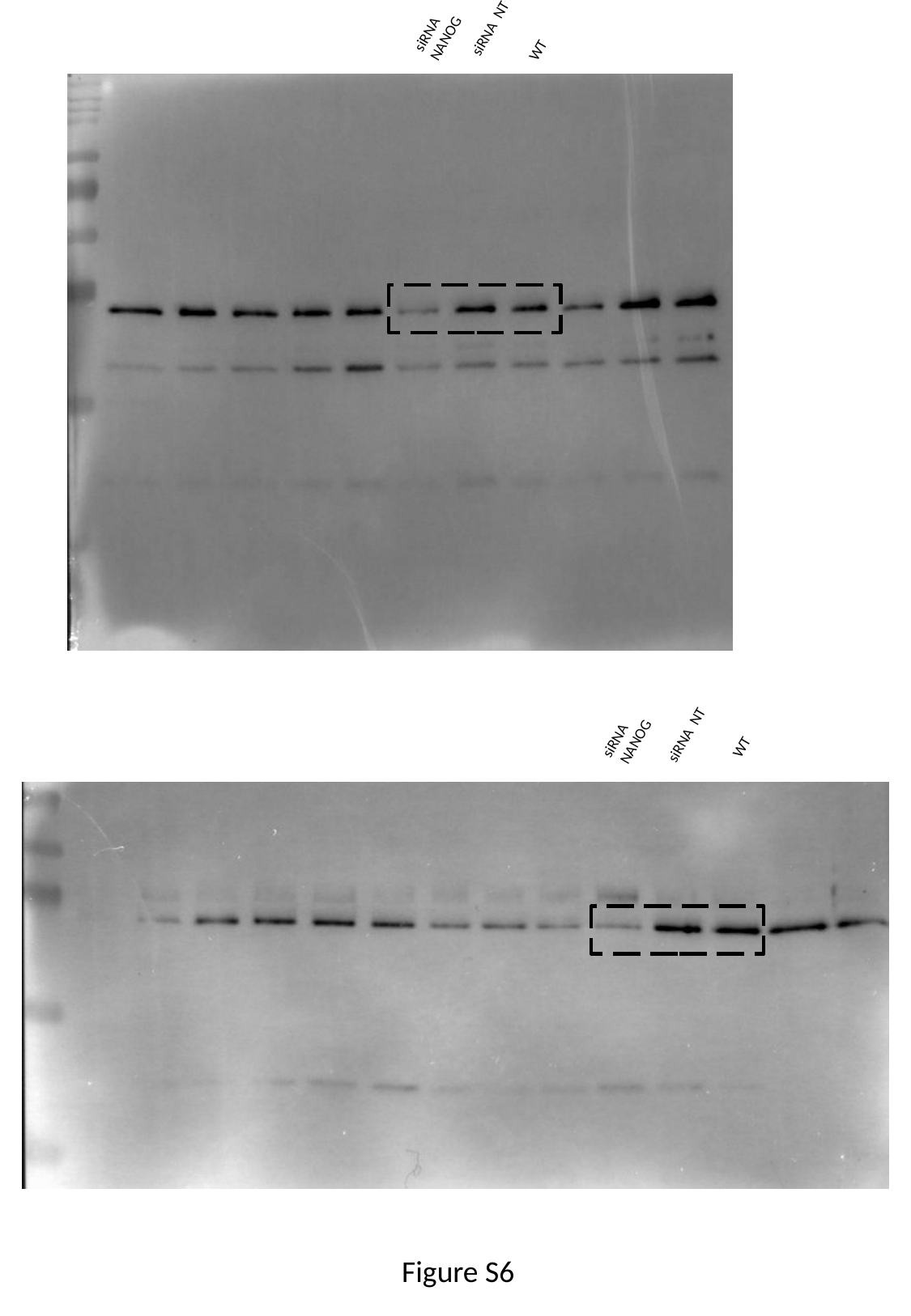

siRNA
NANOG
siRNA NT
WT
siRNA
 NANOG
WT
siRNA NT
Figure S6
